# Research Review: Emanuel Miller Memorial Lecture 2012 – Neuroscientific studies of intervention for language impairment in children: interpretive and methodological problems

**DOI:** 10.1111/jcpp.12034

**Published:** 2013-01-02

**Authors:** D V M Bishop

**Affiliations:** Department of Experimental Psychology, University of OxfordOxford, UK

**Keywords:** Intervention, neuroscience, language impairment, brain imaging, fMRI, ERP, MEG

## Abstract

**Background:**

Our ability to look at structure and function of a living brain has increased exponentially since the early 1970s. Many studies of developmental disorders now routinely include a brain imaging or electrophysiological component. Amid current enthusiasm for applications of neuroscience to educational interventions, we need to pause to consider what neuroimaging data can tell us. Images of brain activity are seductive, and have been used to give credibility to commercial interventions, yet we have only a limited idea of what the brain bases of language disorders are, let alone how to alter them.

**Scope and findings:**

A review of six studies of neuroimaging correlates of language intervention found recurring methodological problems: lack of an adequate control group, inadequate power, incomplete reporting of data, no correction for multiple comparisons, data dredging and failure to analyse treatment effects appropriately. In addition, there is a tendency to regard neuroimaging data as more meaningful than behavioural data, even though it is behaviour that interventions aim to alter.

**Conclusion:**

In our current state of knowledge, it would be better to spend research funds doing well-designed trials of behavioural treatment to establish which methods are effective, rather than rushing headlong into functional imaging studies of unproven treatments.

## Introduction

Over the past few decades, there has been a staggering increase in our ability to visualize the structure and function of the developing brain. In the 1970s, the only option for studying brain structure in a living child was through the murky images generated by the new technique of computerized tomography (Filler, 2009). A handful of experts dabbled in electroencephalography (EEG) for measuring brain function (e.g. John et al., 1980), but cost and technical complexity put such methods out of the reach of the average researcher. Nowadays, neuroscientific methods are included as a matter of course in studies of children with neurodevelopmental disorders. The techniques include functional magnetic resonance imaging (fMRI), near infrared spectroscopy, high-density EEG and magnetoencephalography (MEG) (Frith & Frith, 2008). Our understanding of brain development has been transformed by spectacular image sequences, such as those showing regional loss of grey matter with age (Giedd & Rapoport, 2010). There is a widespread belief that an improved knowledge of brain structure and function will lead to more effective intervention, and it is commonplace to find researchers justifying their studies on this basis. I shall take a critical look at these trends and argue that in no case has neuroimaging influenced the nature or application of intervention for children’s language disorders. The problems with this field of research are twofold: interpretive and methodological.

## Overinterpretation of neurobiological studies of intervention

Let us imagine for a moment that we have a well-designed study that included measures of brain as well as behaviour, and used appropriate analyses to show that a control group and a language intervention group differed after intervention. As Coltheart and McArthur (2012) have noted, brain data cannot tell us whether an intervention is effective: the critical test is whether it changes behaviour. If we see brain changes that are correlated with behavioural improvements, that is of interest in possibly helping us understand the biological underpinnings of change, but we must beware of falling into the trap of assuming that the brain change is somehow more real and meaningful than behaviour change. Ultimately, the goal of intervention is to improve a person’s cognitive or emotional state: for this purpose, physiological changes are of far less relevance than behavioural indicators of improvement, such as test performance, mood ratings or behavioural observations.

Those offering commercial interventions have been quick to pick up on the allure of neuroscience, and there is a host of ‘brain training’ programs available on the internet. Since the brain is the organ that is responsible for language, any intervention designed to improve language can be described this way. In a similar vein we find the new field of educational neuroscience replete with statements such as this one from a recent conference flier: “ The brain is ‘plastic’, according to recent findings in neuroscience, and that concept can help teachers and educators improve learning …. The meeting will focus on the discovery that the brain is not ‘hardwired’ from birth, but holds a remarkable lifelong power to change”. Essentially, saying the brain is plastic and not fixed boils down to saying that children can learn new things – hardly a remarkable finding. Potentially, it could be useful if neuroscience could provide insights into issues such as optimal timing of particular kinds of teaching: e.g. is it better to learn a second language in early childhood than in adolescence? However, although studies of brain development might help explain the neuroanatomical basis for changes in plasticity, the key evidence for plasticity would come from *behavioural* studies comparing how well children of different ages learned (Bruer, 1997).

**Figure 1:**
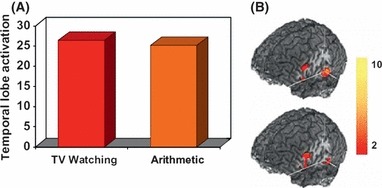
Examples of (A) bar graph and (B) brain image used for the article entitled, ‘Watching TV is Related to Math Ability’, in which watching television and completing arithmetic problems led to similar levels of temporal lobe activation. Reproduced with permission from: McCabe and Castel (2008). Seeing is believing: The effect of brain images on judgments of scientific reasoning. Cognition, 107 (1), 343–352. doi: 10.1016/j.cognition.2007.07.017

Abandonment of critical faculties in the face of neuroscientific evidence has become such a problem that it has become a subject for study in its own right. In a study entitled ‘The seductive allure of neuroscience explanations’, Weisberg, Keil, Goodstein, Rawson, and Gray (2008) found that people were worse at distinguishing good from poor explanations of psychological phenomena when an irrelevant statement about brain function was added. In another study, McCabe and Castel (2008) presented people with illogical articles and found that they more readily accepted them if a brain image was presented. For instance, participants were told of a fictitious study showing that TV watching and completing arithmetic problems both led to activation in the temporal lobe, with the conclusion that watching television improved maths. The similarity in activation was depicted (A) in a bar graph, (B) in a brain image (see Figure 1) or (C) was explained only in the text. Participants were more likely to rate the article as having good scientific reasoning if the brain scan image was included.

One might imagine that neuroscientists should be immune to the persuasive powers of brain images, but this does not seem to be the case. My initial goal in writing this review was to evaluate the contribution of neuroimaging (fMRI, electrophysiological and magnetoencephalographic) studies of language-based interventions. However, when I came to read the relevant literature, I found it difficult to draw any firm conclusions because of methodological failings. There was a consistent pattern of problems that cropped up in study after study. The impression was that editors and reviewers are inclined to overlook weaknesses in research design and analysis if a study involves images of brains or brain activity.

## Studies showing brain changes after intervention: methodological considerations

Studies were found via a literature search on Web of Science for 2003–2011 using topic keywords ‘language’, ‘child*’, ‘brain’ and ‘intervention’ OR ‘remediation’. This yielded over 250 references, but only six met the criteria of reporting data on measures of brain function in children before and after intervention that focused on improving language skills.

### FastForword and fMRI I

Temple et al. (2003) studied a group of 20 children with dyslexia, who underwent fMRI while doing a reading-related task. The same fMRI procedure was carried out before and after computerized language intervention, FastForword. Over the same interval, a comparison group of typical-reading children also took part in two fMRI sessions. Both groups completed a battery of language and literacy tests before and after intervention. The test scores of the dyslexic group improved after the intervention, and they also showed increases in left-hemisphere activation.

### Earobics and ERP

Hayes, Warrier, Nicol, Zecker, and Kraus (2003) considered the impact of a commercially available programme, Earobics (Diehl, 1999), on cortical and brainstem auditory event-related potentials (ERPs) in 27 children. They included an untrained control group, consisting of (a) 15 cases who chose not to participate in the training programme, or who were enrolled after the training programme had begun, and (b) seven typically developing untreated children. The authors focused on the difference in the cortical response to one stimulus (/ga/) between pre- and posttesting on point-to-point *t*-tests, which was significant for the trained subjects but not for the untrained subjects. They concluded that brief auditory perceptual training can influence cortical representation of speech.

### Phonological and motor training group interventions and MEG

Pihko et al. (2007) studied 16 children diagnosed with a developmental disorder of speech and language, who were divided into two groups, matched on age, gender and language background. The first group received an intervention designed to improve phonological discrimination and awareness for 20–30 min three times a week for 8 weeks. The second group received motor training for an equivalent period. Brain responses to syllables were measured in both groups using MEG before and after the intervention period. Behavioural discrimination of the syllables was also assessed. A complex pattern of results was interpreted as indicating that intervention leads to plastic changes in the brain activity of auditory cortex.

### FastForword and fMRI II

Gaab, Gabrieli, Deutsch, Tallal, and Temple (2007) reported an analysis of the impact of the FastForword intervention on fMRI responses to nonlinguistic stimuli that included rapid or slowed frequency transitions. The participant group overlapped substantially with those in the Temple et al. (2003) study and included 22 children with language and/or literacy problems, all of whom received training. An untrained typical-reading group had two scans over the same interval. The analysis focused on brain regions that showed greater activation to rapid versus slowed frequency transitions while participants rated another aspect of the stimuli, frequency. An intervention effect was claimed on the basis that the left prefrontal region, which was specifically activated by rapid transitions in typical readers, showed an increase in activation from pretraining to posttraining in the trained children.

### FastForword and ERP

Stevens, Fanning, Coch, Sanders, and Neville (2008) conducted an electrophysiological study of eight children with specific language impairment (SLI) aged from 6 to 8 years. They concluded that neural mechanisms of selective auditory attention can be enhanced by training, leading to improved language skills. Children received the FastForword training programme for 100 min per day, for 6 weeks. Twelve typically developing (TD) children also received training, and a further 13 TD children received pre- and posttesting of language but no training. Auditory ERPs were available for seven children with SLI, nine of the trained TD children, and 11 of the untrained TD children. During the recording, children listened to stories presented through speakers situated to their left or right. They were instructed to attend to one side, and electrophysiological responses were measured to probe stimuli (‘ba’ or a buzz) occurring on that side. The analysis focused on the difference in the mean amplitude of the ERP in the interval 100–200 ms post stimulus onset (collapsing across syllables and buzzes) between attended and unattended stimuli. The authors argued that there was evidence for differential change in the auditory ERP across the three groups, although the main effect that they reported showed only a nonsignificant trend (*p* < .1).

### Narrative generation, N400 responses and ERP

Popescu, Fey, Lewine, Finestack, and Popescu (2009) studied an electrophysiological component, the N400, which is seen when an incongruous word completes a sentence. For instance, the child might hear ‘When it’s cold, Dad will wear his (pause) ball.’ The brain response to this incongruous completion is contrasted with that seen to an expected final word such as ‘coat’. Typically, one sees enhanced negativity (N400) to the incongruous word. This study was designed to see whether an intervention that involved generation of narratives would lead to enhancement of the N400. Children with language impairments had 10–12 sessions of intervention over a 5-week period. ERP data were available for eight children who had been tested before and after training. All children had poor language skills and five met criteria for SLI (nonverbal IQ of 85 or above). The authors reported that after intervention there was a dramatic reduction in the N400 to congruous words. However, they also noted a lack of correlation between ERP changes and behavioural gains, as well as the possibility that repeated testing could be responsible for some of the ERP changes.

#### Methodological issues

In Table 1, each study is evaluated against specific methodological criteria that are described more fully below. The criteria are mostly based on an attempt to apply design principles from clinical trials (Altman et al., 2001) to educational interventions (Bishop, 2008). They also cover some further issues specific to neuroimaging studies. There is an inevitable subjective element to this evaluation: in general, the aim was to avoid giving a negative rating for minor lapses, but only use this rating for more serious flaws that compromised the conclusions of the study.

**Table 1:** Methodological criteria for evaluating intervention studies, applied to the following studies: (1) Temple et al., 2003; (2) Hayes et al., 2003; (3) Pihko et al., 2007; (4) Gaab et al., 2007; (5) Stevens et al., 2008; (6) Popescu et al., 2009

Criteria	1	2	3	4	5	6
Participants: clinical						
(a) Sample gives adequate power	✓	✓	**x**	✓	**x**	**x**
(b) Appropriate, objective criteria	✓	✓	✓	✓	✓	✓
Random/matched clinical controls	**x**	**x**	✓	**x**	**x**	**x**
Typically developing comparison group	✓	✓	**x**	✓	✓	**x**
Information on dropouts	**x**	**x**	**x**	**x**	✓	**x**
Intervention: adequately described	✓	✓	✓	✓	✓	✓
Outcome measures						
(a) Primary outcomes specified	**x**	**x**	**x**	**x**	**x**	**x**
(b) Reliable, standardized (behavioural)	✓	✓	✓	✓	✓	✓
(c) Measurement blind to group	**x**	**x**	**x**	**x**	**x**	**x**
Reporting of results						
All key data (*N*s, means, *SD*s) reported	**x**	✓	✓	✓	✓	**x**
Data analysis						
(a) Intervention effect appropriately analysed	**x**	**x**	✓	**x**	**x**	**x**
(b) Correction for multiple comparisons; no ‘double-dipping’	**x**	**x**	**x**	**x**	**x**	**x**

Key: ✓: mostly meets criterion; x: fails criterion.

*Intervention and principal methodology*: (1) FastForword and fMRI (Temple et al., 2003); (2) Earobics and ERP (Hayes et al., 2003); (3) Phonological and motor training group interventions and MEG (Pihko et al., 2007); (4) FastForword and fMRI (Gaab et al., 2007); (5) FastForword and ERP (Stevens et al., 2008); (6) Narrative generation and ERP (Popescu et al., 2009).

#### Participants: clinical group

Bishop (2008) noted three key aspects of sample selection. First, participants should be identified by objective criteria; second, they should be comparable with those for whom the intervention is intended and third, the sample size should give adequate power to detect an intervention effect. Overall, the six studies featured here did reasonably well in terms of using objective and appropriate criteria to select clinical cases. Power was, however, weak in all cases and seriously limited for three of the studies, with fewer than 10 children in the intervention group. With 10 participants in a repeated measures design, the power to detect an effect size of 0.5 is only .42, with *p* < .05 on a one-tailed test. Underpowered studies can be worse than no study because they may lead one to conclude there is no effect of intervention when in fact a clinically useful effect may be present.

#### Participants: control group

**Figure 2:**
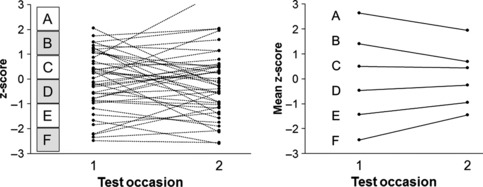
Simulated data to illustrate regression to the mean. The left hand plot shows individual data points, simulated to have correlation of .5 between time 1 and time 2. The panel showing A, B, etc. shows the ranges of time 1 scores for which mean z-scores are shown in the right-hand plot. The overall mean does not change from time 1 to time 2. However, the means for time 2 increase for those with initial low scores (E and F) and decrease for those with initial high scores (A and B): this is regression to the mean, and is an inevitable consequence of imperfect test–retest reliability

A key question is whether changes after intervention are due to the intervention. There are many other reasons why change may be seen. Many cognitive tests show practice effects, i.e. improvement simply as a consequence of having done the test before (McArthur, 2007). Some improvement may be due to maturation. For instance, Summers, Larson, Miguel, and Terrell (1996) found that language screening test scores in a population sample of 5-year-olds improved by almost one SD when children were retested after 7 months, even though no language intervention was given and the scores were age-standardized. Improvement can also be due to regression to the mean (Campbell & Kenny, 1999). This refers to the statistical artefact that occurs when the same test is used both to select low-scoring individuals and to evaluate their progress (Zhang & Tomblin, 2003); it is inevitable when a measure has less than perfect reliability (see Figure 2). All these potential confounds can be controlled for by including a control group that is comparable which the intervention group but which does not receive the target intervention. To ensure that allocation of participants to intervention or control groups is unbiased, potential participants should first be selected to meet study criteria, and then assigned at random to one group or the other. Sometimes explicit group matching is used, although this can be problematic as it is difficult to match on all relevant variables. Typically, in contemporary randomized controlled trials, the control group is not untreated, but rather receives an alternative intervention (Evans, Thornton, Chalmers, & Glasziou, 2011). This allows one to also control for potential placebo or expectation effects. For instance, in a study designed to improve language skills, a control group could receive a maths or motor skills intervention.

Many authors appear to regard it as implausible that changes could arise without intervention; consequently they assume that a treatment effect can be demonstrated by comparing pre- and postintervention scores of a treated group. However, adequately controlled behavioural studies demonstrate just how dangerous this assumption is. For instance, a meta-analysis of FastForword found no significant benefits of the intervention in studies where adequate controls were used (Strong, Torgerson, Torgerson, & Hulme, 2010). Typically in these studies, the treated group improved, but so did the control group. This illustrates the inadequacy of concluding that an intervention is effective on the basis that *t*-tests show significant gains on language measures after intervention (cf. Temple et al., 2003). Perhaps even more unexpected are changes seen on fMRI on repeated testing, as exemplified in a spelling intervention study by Gebauer et al. (2012). Over a 5-week interval, children showed increased activation in the precuneus regardless of whether or not they had intervention. Furthermore, an untreated group of poor spellers showed increased activation in right lateral occipital cortex and right middle temporal cortex, and an untreated typically spelling group showed increased activation in bilateral middle temporal and occipito-temporal regions over the same time period.

Of the six studies in Table 1 only that by Pihko et al. (2007) had a suitable control group for estimating the effect of the intervention. Four studies (#1, #2, #4 and #5) included other comparison groups, e.g. typically developing children tested at pre- and posttest. This does not adequately control for effects of practice and maturation because the comparison group is likely to differ on initial measures, both behavioural and brain based. Typically developing children may show little change on behavioural tests compared with language-impaired children because there is less room for improvement.

A typically developing comparison group can, however, be useful for answering a related question, namely whether successful intervention is associated with normalization of brain function. If there is a significant interaction, such that a significant difference between a clinical and control group reduces or disappears postintervention, then this is suggestive of a treatment effect. As Dichter, Sikich, Song, Voyvodic, and Bodfish (2012) noted, however, without a clinical control group it can be hard to rule out placebo effects or differential stability of results in the two groups – possibly due to more anxiety- or movement-related artefact in the clinical group. A significant correlation between behavioural improvement and normalization of brain measures, however, would support the idea that the brain change indexes the underlying mechanism that mediates the behavioural change.

#### Dropouts

In many intervention studies, some participants drop out before the study is completed. This may be for logistic reasons, such as illness or relocation, but it can also arise if the participant does not feel that the intervention is effective, or if, conversely, they do not think they need the intervention because they are doing so well. This means that there is potential for bias when assessing interventions if dropouts are ignored. In particular, beneficial effects will be overestimated if the only people who stay in the study are those who make good progress. It is important to report on dropouts so that one can establish whether such bias is likely to affect results. Only one of the six studies under consideration reported information about dropouts.

#### Intervention

Interventions have to be clearly enough described for other researchers to replicate the study. Four of the studies in Table 1 used commercially available intervention programmes that are in the public domain, so that others could potentially replicate the study. The other two studies used experimental interventions which were described in reasonable detail.

#### Outcome measures: (a) Primary measures specified

One point that has become standard in the field of clinical trials is the need to distinguish between primary and secondary outcomes (Freemantle, 2001). Typically, when running a behavioural intervention, one wants to do a fairly detailed assessment, rather than focusing on just a single outcome measure. Indeed, there may be particular interest in the question of whether children with a specific profile fare better or worse with the intervention. The problem, though, is that if a battery of, say, eight measures is used on pre- and posttest, then the likelihood of one measure showing a statistically significant effect by chance is higher than if a single measure is used. Post hoc division of a sample into subgroups, on the basis of initial exploration of the data, is particularly likely to generate false-positive findings (Freemantle, 2001). This does not preclude exploratory data analyses, but post hoc findings should be regarded as hypothesis generating (i.e. needing replicating in a new sample), rather than hypothesis testing.

This can be a particular issue in brain-based measurements, which typically lead to a huge number of potential measures, and all the studies in Table 1 were problematic in this regard. For instance, in their MEG study, Pihko et al. (2007) conducted several analyses of variance looking at both strength and latency of different evoked components in left and right hemisphere at two time points. The authors stated that the intervention influenced the amplitude of the mismatch responses of the phonological intervention group, because the strength of the response to one deviant in one condition and one hemisphere was significantly enhanced after intervention. However, their analytic approach is likely to generate spurious findings unless care is taken to correct for the many statistical comparisons (see Data analysis, below).

#### Outcome measures: (b) Reliability and standardization

A good study will use standardized behavioural instruments for assessing participants’ abilities before and after treatment. In the English-speaking world there are well-established standardized assessments of language and literacy that are reliable, sensitive and valid, and selection of behavioural assessments was one aspect of methodology that was generally satisfactory in the studies reviewed here.

Neuroimaging measures, however, have very different characteristics. Unlike standardized cognitive tests, for most brain measures we do not know what is normal and abnormal at a given age. Also, it is only relatively recently that much attention has been paid to reliability of brain imaging data, i.e. the extent to which results would replicate on retesting (Bennett & Miller, 2010; Dichter et al., 2012). It is easy to fall into the trap of assuming that because the brain is a physical organ, measurements from it will be consistent unless some specific training has taken place. However, functional measures are bound to vary from one occasion to another, and test–retest reliability can vary substantially from region to region (Caceres, Hall, Zelaya, Williams, & Mehta, 2009). The method of analysis may also affect reliability. A recent concern has been raised about measures of functional connectivity, which can be substantially influenced by head motion (Power, Barnes, Snyder, Schlaggar, & Petersen, 2012), something that may decline with experience in the scanner, and is likely to vary between clinical and control groups (Eloyan et al., 2012). Even with structural measures from MRI, small changes in image orientation and magnetic field instability can influence measurements (Morey et al., 2010).

The other methods featured in Table 1, EEG and MEG, involve indexing moment-by-moment neuronal activity. Here too there has been little attention to measurement issues that are taken for granted in psychological assessment. To take one example, the mismatch negativity (MMN), an electrophysiological index of change detection, has been used to index auditory discrimination in children with language and literacy problems (Bishop, 2007a). Although it has been recommended as a clinical diagnostic tool (Näätänen, 2003), its reliability and validity are too low for this purpose, even in compliant adults given a long series of trials (e.g. Bishop & Hardiman, 2010). Data from MMN may be useful in group comparisons, but one needs to be cautious in interpreting changes in MMN, which can simply arise because of noisy data.

#### Outcome measures: (c) Blind assessment

One measure of quality of randomized controlled trials is blinding, i.e. ensuring assessments are conducted by people who are unaware of the treatment group that the participants came from, to minimize bias (Schulz & Grimes, 2002). This can be difficult to arrange in practice, and is seldom done except in medical contexts. None of the studies reviewed here mentioned blind assessment, consistent with a general assumption by psychologists that standardized instruments use objective protocols, which should guard against biased results. Furthermore, analysis of fMRI data is largely automated. Nevertheless, the literature on randomized controlled trials suggests that psychologists and neuroscientists should be less sanguine about this issue, as there is evidence of a potential risk of bias for any measure, either behavioural or neuroscientific, that involves a degree of subjective judgement (Ioannidis, 2011). In general, medical trials that use blinding obtain smaller treatment effects than those that do not, even when the measures involve little subjective judgement (Day & Altman, 2000). Furthermore, there is ample scope for biasing results if the researcher’s judgement is used to determine, for instance, which brain regions to investigate, or which thresholds to use, after inspecting data for treatment and control groups.

#### Reporting of results

Simmons, Nelson, and Simonsohn (2011) raised a serious concern about lack of replicability in psychological studies, coining the term ‘false positive psychology’ to describe research that finds significant effects by post hoc selection of variables to analyse from a large array of possibilities. Carp (2012) noted that fMRI is particularly susceptible to this bias because of the flexibility in methods of data analysis it typically allows. One reason why the medical trials literature requires advance specification of primary outcome measures (see above) is to avoid misleading results that may arise through data dredging. In addition, it is important to report all relevant data (if necessary, as supplement material), and effect sizes and confidence intervals should be given.

Many studies report only positive findings, without mentioning other variables and analyses that were used. This can be a particular problem for articles that appear in journals with very strict word limits. For instance, Temple et al. (2003) gave means and *SD*s for key behavioural measures, but other variables, not previously mentioned, were included in a correlational analysis, and it is impossible to judge how many variables were tested altogether. For the task done in the scanner, mean correct responses were reported for the two groups, but standard deviations were not given and reaction time data were missing. No data were reported on fMRI changes in the typically developing group from the first to second scan, although this would have been useful in giving an indication of reliability of these measures.

#### Data analysis: (a) Analysis of intervention effect

The effect of intervention should be assessed using methods that explicitly compare control and intervention groups. Demonstration of a significant interaction between testing occasion (pre- vs. postintervention) and group (experimental vs. control) can be used to show a treatment effect if groups are well-matched prior to intervention. Analysis of covariance, comparing posttest scores for two groups while covarying pretest scores is preferable, as it adjusts for initial level of performance, optimizing power for finding a group difference. Unfortunately, many studies adopt neither approach. In a review of neuroscience studies, Nieuwenhuis, Forstmann, and Wagenmakers (2011) noted a widespread failure to perform appropriate analyses when looking for differential effects of an experimental manipulation in two groups. Their general point was that researchers often focus on differences in significance levels (e.g. Group A improves significantly from time 1 to time 2, whereas Group B does not), when they should be considering the significance of differences (i.e. is the change from time 1 to time 2 reliably greater in Group A than in Group B).

Clearly, studies cannot meet this methodological criterion if they did not have an appropriate control group, as was the case for five of the six studies. As noted above, behavioural measures may change substantially from a first to second testing, even if no intervention occurs, and so cannot be interpreted as showing an intervention effect. Nor is this difficulty solved by demonstrating that a treated group changes and a comparison group does not. It is particularly misleading if researchers compare the significance of change scores in two groups when the groups differ in size, as was done by Hayes et al. (2003), because the same amount of change will be more likely to be statistically significant in large sample than a small sample.

What of brain imaging results? The way in which imaging data are presented exacerbates the tendency to overinterpret differences within or between groups. Typically, results from fMRI are shown on a diagrammatic brain, with ‘blobs’ indicating regions where a group of individuals shows significant activation in one condition relative to another. The location and size of blobs are dependent on decisions about thresholding, which can make a small difference between groups look like an all-or-none effect. Furthermore, this type of representation does not depict the variation within the group. It is easy to be misled into thinking that all participants show a similar level of activation, and that the overall image is representative of individuals in the group. This is not necessarily true. There is a range of possible explanations for a failure of a group to show activation on fMRI in a given brain region. It may be that the brains of these children are unresponsive to the critical task dimension. Alternatively, there may be strong levels of activation, but with high within-group variation, either in level or in location of activation. These possibilities cannot be distinguished if one relies solely on an image of significant areas of activation when comparing two groups. And again, the problem is compounded if group sizes are unequal.

**Figure 3:**
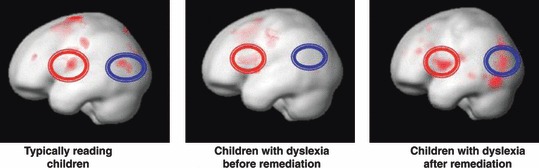
Brain activation differences in dyslexia and its treatment, based on data from Temple et al. (2003). Figure and explanatory legend (below) reproduced with permission from Gabrieli, J. D. (2009). Dyslexia: a new synergy between education and cognitive neuroscience. Science, 325 (5938), 280–283. ‘Functional magnetic resonance imaging activations shown on the left hemisphere for phonological processing in typically developing readers (left), age-matched dyslexic readers (middle), and the difference before and after remediation in the same dyslexic readers (right). Red circles identify the frontal region, and blue circles identify the temporo-parietal region of the brain. Both regions are hypoactivated in dyslexia and become more activated after remediation’

**Figure 4:**
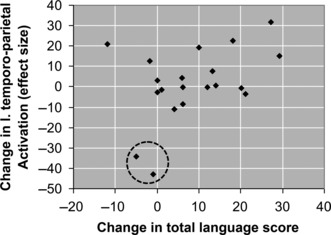
Scatterplot based on Temple et al. (2003), showing relationship between change in Total Language Score and change in left temporo-parietal activation, from pre- to posttraining in dyslexic children. The dotted circle indicates two outliers who show a decrease in activation over this interval. With these participants included, the Pearson correlation is *r* = .41, two-tailed *p* = .06. With the two outliers excluded, *r* = .24, *p* = .33

The potentially misleading nature of imaging data is illustrated by Figure 3, from Temple et al. (2003). Although they did not include an untreated clinical group, they did have data on a typically developing comparison group, and so potentially could consider whether the brain responses of dyslexic children normalized after intervention. It was argued that they did because after intervention the dyslexic children showed increased activation in brain regions that had previously been underactive, and which were activated in the typically developing children from the outset. These conclusions, however, were based on a questionable analysis. In effect, the researchers were claiming an interaction effect, i.e. a difference between groups on the first scan which reduced at the second (posttraining) scan, but this was not tested statistically by testing for an interaction with group. Instead, it was argued that the changes seen in dyslexic brains make them look more like typically reading children on the basis of the brain maps (see Figure 1), which do not depict the variation within groups. Furthermore, the analysis focused on change in activation from pretraining to posttraining within the dyslexic group, after the *average* change in activation of the typically reading group had been subtracted from the dyslexic activations. The justification for this approach is that it lets us see changes in activation that were specific to the dyslexic group, but this is invalid. What is needed is a statistical comparison that takes into account variability within *each* group. We can gain an impression of within-group variation from the scatterplot reported by Temple et al. (2003) showing the distribution of a measure of change in left temporo-parietal activation in the dyslexic group, and it is evident that this is substantial, with two individuals showing large decreases, and many others showing no change (see Figure 4).

Similar issues arise in reporting of electrophysiological data, where it is common to report results as grand mean waveforms. It is not possible to tell whether two waveforms are reliably different without information on the variability within each group. This was a limitation of the study by Popescu et al. (2009), who showed grand average waveforms and results of statistical tests, but did not report means, SDs or effect sizes for ERP measures.

#### Data analysis: (b) Correction for multiple comparisons

Bishop (2007a) noted that in electrophysiological studies investigators have many possible ways of looking at the data, by examining different peaks, different methods of identifying peaks, different electrodes, different time windows and so on. Given such a huge range of options, if you look at any data set in enough ways, you will find a comparison that is significant at the .05 level: the problem is that the significance levels are only meaningful if the analysis was specified in advance, before the data were inspected. In the field of structural and functional MRI, this problem is magnified, with data available from thousands of voxels. The field has evolved procedures for correcting for multiple comparisons, but there is debate over the specifics of how this should be done, and variation in the methodology employed (Bor, 2012).

Correlations are often used to support analysis of main effects in intervention studies, and to identify characteristics of those who respond to intervention. They are, however, susceptible to spurious findings if large numbers of comparisons are conducted in an unconstrained way. Temple et al. (2003) reported some significant correlations between behavioural measures and selected brain regions of interest. However, given the large number of brain regions considered and the range of behavioural measures (which included measures taken from the training package, and a phonological processing measure that was not described in the Methods section), spurious correlations are likely. It is not stated how many correlations were computed, but it is clear that the results would not survive correction for multiple testing: The correlation given most prominence, between language improvement and increase in left temporo-parietal activation, was significant at .05 level only on one-tailed test. It falls close to zero if we take out two outliers who showed a *decrease* in activation coupled with a lack of language improvement (see Figure 4).

‘Double dipping’ is a dubious procedure that is often used with the aim of ameliorating the problems of multiple comparisons. It refers to a two-stage analysis where a data set is first inspected to identify a subset of interest (e.g. a set of voxels, a specific electrode, a time region), and then statistical analysis is confined to that subset of the data (Kriegeskorte, Simmons, Bellgowan, & Baker, 2009). This is likely to yield spurious positive findings unless the results that are analysed are independent of the selection criterion. This is because dependent variables are not pure measures of an effect of interest: they also contain random error. The notion of a measure having a ‘true score’ component and an ‘error’ component is well established in psychometrics (Lord & Novick, 1968), but has much broader application. Suppose I have groups A and B for whom there are fMRI data on two conditions which differentially activate a given brain region. If I select for analysis the brain region that gives maximal activation for group A, and then compare A and B on this region, the likelihood is that group B will show less activation than A because the maximum score of A incorporates random error as well as true score.

This may be easiest to understand by imagining a situation where a score is determined *only* by chance, such as when two people pick three cards from a pack at random. We repeat this with 10 different packs of cards (analogous to brain regions), and then decide to allocate a prize to the person who gets the highest scoring cards with a given pack. But there is a catch. We select the pack that person A got their highest score on. Clearly this is unfair because this biases the outcome in favour of person A. Of course, in neuroimaging contexts, data are not determined just by chance, but the point raised by Kriegeskorte et al. (2009) is that there will be error associated with any measure, and this means that this kind of bias will creep in to any analysis where there is a lack of independence between the process of selecting a variable and analysis of that variable.

Consider, for instance, the approach adopted by Gaab et al. (2007), who compared pretraining activation for dyslexic and typical readers by identifying regions that showed more activation to rapid than slow transitions in the latter group. They found that the dyslexic group showed less of an effect of transition frequency for these regions. However, this is directly analogous to the playing cards example and the result is misleading because it does not take into account bias introduced by measurement error.

## Overview of studies of neurobiological impact of intervention

The summary of methodological features in Table 1 highlights strong commonalities between studies in terms of their strengths and weaknesses. Studies in this area use psychometrically strong measures to select and evaluate language skills in their participants. Interventions are mostly clearly described. But there are consistent methodological flaws.

1. Researchers embark on studies of neurobiological impact of interventions without first checking how effective the intervention is. Without data from randomized controlled trials showing a significant intervention effect, it seems a waste of research funds to do expensive and difficult studies on the neurobiological impact.
2. Sample sizes are usually small, and some studies were seriously underpowered. This is understandable: it can be difficult to recruit children for studies that involve intensive training and electrophysiological or neuroimaging procedures. Nevertheless, it means that we can conclude very little if the data do not show any effect of intervention because only large effects would be detectable. Sample sizes should be determined by a consideration of the effect sizes associated with the treatment in prior studies. If we assume a typical treatment effect size of around .5, then for 80% power on one-tailed test at .05 level we would need a total of 102 participants divided into treatment and control groups (Faul, Erdfelder, Lang, & Buchner, 2007). It may be that the only way to achieve adequate numbers will be to organize multicentre trials.
3. Another striking feature of these studies is the failure to include a control group that is equivalent to the intervention group. There appears to be a lack of awareness of potential confounds and a widespread assumption that any changes that seen from pretest to posttest can therefore be attributed to the intervention.
4. The problem identified by Henson (2005) of inappropriate statistical analysis pervades this field. Typically, researchers would conclude that they had demonstrated an intervention effect because they found a statistically significant difference in a trained group and not in an untrained group. There appeared to be little recognition of the fact that sample size as well as effect size will determine statistical significance, and sometimes this analytic approach was adopted even with unequal-sized groups.
5. Data dredging without any constraint from a priori predictions was common. There is growing recognition that this is a particular problem in neuroimaging studies (e.g. Ioannidis, 2011). The related problem of using an initial scrutiny of the data to decide which variables to assess, or ‘double-dipping’ (Kriegeskorte et al., 2009), was also in evidence.

There seems to be little awareness of the extent of these problems. Consider the Temple et al. (2003) study from Table 1. This was published in a top journal and at the time of writing has had 284 citations in Web of Science. I took a random sample of 50 of these and found that all but one of them repeated the authors’ conclusion, i.e.: FastForword is effective, it increases activation in specific regions of brains of dyslexic children, the brains of dyslexic children become more like those of typical readers and this brain change is correlated with language improvement. Yet critical appraisal of this study taking into account its methodological shortcomings suggests that none of these conclusions is supported.

Many of the methodological problems highlighted here affect intervention studies of all kinds and are not specific to neuroimaging studies. Nevertheless, they do appear to be particularly common in intervention studies that use neuroimaging methods, and it is worth speculating as to why this is so. In the introduction I suggested that scientists, like the general public, may be captivated by the rich information and attractive images that neuroimaging yields, to the extent that they forget the importance of asking questions about issues such as reliability and within-group variation. Another reason may be a mismatch of scientific culture: quite simply, those using neuroimaging methods may be unaware of the large literature on design and analysis of clinical trials. Clinical trials methodology has developed gradually, and it has taken many years to recognize the biases that can creep into intervention studies and distort conclusions (Evans et al., 2011). In addition, the neuroimaging studies reviewed here were beset by specific analytic problems that have only received attention in recent years, and that reflect problems in responding appropriately to the surfeit of data that these methods generate (Poline & Poldrack, 2012).

## How might neuroimaging studies influence intervention in future?

It is early days for brain-based studies of intervention. Potentially, fMRI studies can show us which brain regions are involved in behavioural change. They can also indicate whether an effective intervention makes the brains of treated children more like those of typically developing peers, or whether they use different brain regions in a compensatory fashion. A demonstration that neurobiological differences between an impaired and typically developing group are reduced or eliminated by effective treatment would provide powerful evidence that the brain circuits involved are implicated in aetiology of the disorder, rather than just coincidental findings. Thus, neurobiological information can complement behavioural evidence in improving our understanding of the underlying nature of training effects (Henson, 2005).

The hope is often expressed that by understanding the underlying brain circuitry of disorder, we may be able to devise better *behavioural* interventions, but this is a far greater challenge. One simple-minded view is that if we know what functions a brain region subserves, then we should be able to train it. For instance, the Dore programme (Dore, 2006) is based on the premise that many developmental disorders, including language and reading impairment, are caused by problems in the cerebellum. Eye–hand coordination and balance functions are known to be mediated by the cerebellum so the idea is that problems with reading and language can be treated by doing physical exercises involving balance and coordination. This is a radically different approach to conventional speech–language therapy or remedial reading, where the therapist works directly on the child’s language or literacy skills. It would be exciting if language problems could be ameliorated without working on language, or reading problems could be helped without any reading practice, but to date there is no scientifically robust evidence for efficacy of such an approach (Bishop, 2007b). We cannot rule out the possibility that knowledge of which brain regions are influenced by training might suggest effective new treatments, but this is highly speculative.

Knowledge of brain functions may, however, come into its own when integrated with intervention approaches that aim directly to alter brain function. I will briefly describe three such approaches: smart drugs, brain stimulation and neurofeedback.

### Smart drugs

Methylphenidate and related drugs have been used for many years to treat attention deficit hyperactivity disorder (ADHD), despite misgivings about using long-term medication with children (Sahakian & Morein-Zamir, 2007). Randomized controlled trials show methylphenidate to be more effective than behavioural interventions for controlling symptoms (Jensen et al., 2001). Information from fMRI, ERP or related methods can help specify the particular pathways that are implicated in effective pharmacological agents, or may even aid discovery of new drug treatments, through knowledge of neurotransmitter characteristics of specific brain systems that change with effective intervention. For instance, a comparison of children with ADHD on and off medication showed that stimulant medication was associated with reduced activity in ventral anterior cingulate cortex while doing a Stroop task (a measure of cognitive inhibition).This was interpreted as indicating suppression of default-mode activity, a neural correlate of mind wandering (Peterson et al., 2009). The pattern of activation on medication became more similar to that of an unimpaired comparison group. This study avoided many of the problems inherent in pre- versus posttreatment comparisons because the drug effect is transient, making it possible to compare children on and off medication in a counterbalanced design.

Findings from brain imaging may also challenge preconceptions about how drugs work. In a structural MRI study of adults with bipolar disorder, Lyoo et al. (2010) found that treatment with lithium was associated with grey matter volume increase, whereas treatment with valproic acid was not, even though both treatments were equally effective in symptom reduction. This challenged the notion that the two drugs had similar mechanisms of action in the brain, although conclusions were tentative because of lack of comparability between groups in sample size and treatment duration.

In the future, we may start to see other pharmacological agents being used to treat a wider range of neurodevelopmental disorders, extending to drugs designed to improve learning. Experimental trials of so-called ‘smart drugs’ have focused on use with adults: either to assess changes in cognitive function in volunteers, or to assess therapeutic use in patients with neurological impairments. As documented by Sahakian and Morein-Zamir (2011), there is growing illicit use of drugs such as modafinil by adults who want to boost their memory, attention and alertness. In principle, if a drug can be shown to boost memory and learning, it may be feasible to use it with children with language problems, particularly if its use is synchronized with intervention sessions. There is precedent for this approach: studies conducted in the 1980s found that the drug piracetam boosted learning in dyslexic children (Wilsher, 1987). However, safety concerns precluded it being licenced.

It is, of course, appropriate to be cautious in use of pharmacological agents for childhood disorders. Most drugs that influence cognitive function also have effects on other aspects of physiology, and it is vital that we do not compromise the health of our children in a quest for treatments for their learning difficulties. In addition, a drug that may be safe for use by adults or animal models could have adverse effects on the developing child. It is unfortunate that the only way to discover long-term effects in children is by trial and error.

### Transcranial direct current stimulation (TDCS)

Both human studies and animal models confirm that application of a very low amplitude current (1–2 mA) via electrodes on the scalp stimulates the brain by modifying cortical excitability (Been, Ngo, Miller, & Fitzgerald, 2007). TDCS is typically applied for around 20 minutes, and its effects persist for up to one hour. The effect of the stimulation will depend on both the location of the electrodes and the direction of current flow. It is thought to work by modifying neural membrane function (Stagg & Nitsche, 2011). It appears to be safe and is increasingly used with adults to treat depression as well as neurological impairments, especially those involving motor function and language (Utz, Dimova, Oppenlander, & Kerkhoff, 2010). TDCS is sometimes confused in the media with electroconvulsive therapy or transcranial magnetic stimulation, both of which are more invasive procedures. TDCS does not involve electric shock, and experimental participants typically report little or no sensation when it is applied; at most, there may be a tingling sensation at the start of the session. Functional imaging studies could be important in developing effective TDCS for neurodevelopmental disorders by providing information about which brain regions are most active during task performance to guide electrode localization. Therapeutic applications would involve applying TDCS during a training session where new learning is encouraged. Nevertheless, despite its apparent safety, at the time of writing, TDCS remains untested in children.

### Neurofeedback

Neurofeedback is a form of biofeedback in which a person is trained to modify their brain waves by visual and/or auditory feedback. The method is increasingly attracting attention as an intervention for ADHD, where the goal has been to modify the frequency spectrum of spontaneous neural oscillations. Computer programs have been developed to analyse the frequency spectrum of resting EEG and use this to control feedback to the participant in a game-like format. Preliminary trials have been inconsistent, but there is some encouraging evidence that this may help children learn to control their attentional state, and so be useful for ADHD (Gevensleben et al., 2009). The possibility of extension to other kinds of specific learning impairment has not been explored, but electrophysiological studies of conditions such as SLI and dyslexia indicate abnormal functioning of some oscillatory mechanisms (Bishop, Hardiman, & Barry, 2010; Hamalainen, Rupp, Soltesz, Szucs, & Goswami, 2012; Kraus, 2012). Studies using ERP or MEG to monitor changes with intervention could be used in future to select specific oscillatory frequencies to target in biofeedback training.

## Conclusion

Page (2006) expressed concern at the way cognitive psychology is increasingly being reduced to a subbranch of neuroscience, fuelled by media obsession with studies that show ‘… when people do X, a part of their brain activates’. He pointed out that basic cognitive psychology studies are increasingly ousted by expensive neuroimaging experiments, with research funders attracted by proposals that include images of the brain. I would argue that a similar process is influencing developmental neuropsychology. The impression is that the field is trying to run before it can walk. Our first priority should be to first develop interventions for children with language impairments and other neurodevelopmental disorders, and to produce good evidence of their efficacy using randomized controlled trials. Second, we also need to do far more methodological work to ensure our neuroimaging tools are as reliable, sensitive and standardized as our behavioural measures (Dichter et al., 2012). Third, we will need to develop multicentre collaborations to do studies with adequate statistical power to detect treatment effects. Only then will we be in a strong position to combine neuroimaging with intervention to answer questions about underlying mechanisms of effective intervention.

**Key points**

- It is popularly believed that studies of brain function will lead to improved intervention for children with neurodevelopmental disorders.
- Limitations of neuroimaging (fMRI, EEG and MEG) data on children are widely underestimated.
- Neuroimagers need to combine their technical expertise with methodological insights from the fields of clinical trials and psychometrics.
- There is potential for neuroscience to inform intervention through application of new methods that aim directly to alter brain function: neuropharmacology, brain stimulation and neurofeedback.
- Well-designed, large clinical trials of behavioural interventions should be a priority – without these, neuroimaging will not be able to fulfil its promise.

## Correspondence

Dorothy V.M. Bishop, Department of Experimental Psychology, University of Oxford, South Parks Road, Oxford OX1 3UD, UK; Email: dorothy.bishop@psy.ox.ac.uk

## Supporting information

**Video S1** Video of Emanuel Miller Memorial Lecture 2012
