# Supplementary material for: Research Review: Emanuel Miller Memorial Lecture 2012 – Neuroscientific studies of intervention for language impairment in children: interpretive and methodological problems
Source: J Child Psychol Psychiatry. 2013 Jan 2;54(3):247–59. doi: 10.1111/jcpp.12034 (PMC3593170; doi:10.1111/jcpp.12034)
Supplement: Supplementary file 1 [file jcpp0054-0247-SD1.docx]

**Supporting information for Bishop: Research Review: Emanuel Miller Memorial Lecture 2012 – Neuroscientific studies of intervention for language impairment in children: interpretive and methodological problems [doi: 10.1111/jcpp.12034]**

Video of Emanuel Miller Memorial Lecture2012, presented by Dorothy Bishop: <https://s3-eu-west-1.amazonaws.com/acamh.recordings/2012/Dorothy+Lowres/Dorothy_lowres.mp4> [234 MB .mp4 file]
